# Retraction Note: *N6*-methyladenosine induced mir-143-3p promotes the brain metastasis of lung cancer via regulation of VASH1

**DOI:** 10.1186/s12943-023-01840-9

**Published:** 2023-08-12

**Authors:** Hongsheng Wang, Qianqian Deng, Ziyan Lv, Yuyi Ling, Xue Hou, Zhuojia Chen, Xiaoxiao Dinglin, Shuxiang Ma, Delan Li, Yingmin Wu, Yanxi Peng, Hongbing Huang, Likun Chen

**Affiliations:** 1 Guangdong Key Laboratory of Chiral Molecule and Drug Discovery, School of Pharmaceutical Sciences, Sun Yat-sen University, Guangzhou, 510006 Guangdong China; 2 Department of Medical Oncology, State Key Laboratory of Oncology in South China, Collaborative Innovation Center for Cancer Medicine, Sun Yat-sen University Cancer Center, Guangzhou, 510060 Guangdong China; 3 Cancer Center, Sun Yat-Sen Memorial Hospital, Sun Yat-Sen University, Guangzhou, 510120 Guangdong China; 4 Department of Medical Oncology, Henan Cancer Hospital, the Affiliated Cancer Hospital of Zhengzhou University, 127 Dongming Road, Zhengzhou, 450008 Henan China; 5 Department of Medical Oncology, Zhongshan City People Hospital, Zhongshan, 528403 Guangdong China

**Retraction note:** ***Mol Cancer*** **18, 181 (2019).**

10.1186/s12943-019-1108-x.

The Editor-in-Chief has retracted this article at the First Authors’s request. After publication, concerns were raised regarding highly similar images in the figures. Specifically:

- Fig, 2F H1975 Con and Fig. 5I VASH1-C169A Con images appear to overlap.
- Figure 4C Vector and Vector + rVEGFA images appear to overlap.
- Figure 7A Control CD31 image appears highly similar to Fig. 6a sh-Mettl3 FN in [1].
- Figure 2F H1975 Con and Fig. 2E Vehicle Con appear to overlap.
- Figure 5E PTX Con and 5I VASH1-C169A Con appear to overlap.
- Figure 5F miR-143-VASH1-C169A 120 min image appears highly similar to Fog. S5C Con 1 µM Noc.

The authors have stated that incorrect images were selected during figure preparation, and that the Fig. 7A Control CD31 image is correct in this article, but was misused in [1].

Due to the high number of errors in the figures, the Editor-in-Chief no longer has confidence in the presented data.

Qianqian Deng, Zhuojia Chen, Xiaoxiao Dinglin, Shuxiang Ma, Yingmin Wu and Hongbing Huang agree to this retraction. Hongsheng Wang has agreed to this retraction but not the wording of this retraction notice. Ziyan Lv, Yuyi Ling, Xue Hou, Delan Li, Yanxi Peng and Likun Chen have not responded to any correspondence from the editor or publisher about this retraction.
